# Supplementary material for: Correcting directional dark field x-ray imaging artefacts using position dependent image deblurring and attenuation removal
Source: Sci Rep. 2024 Aug 1;14:17807. doi: 10.1038/s41598-024-68659-2 (PMC11294358; doi:10.1038/s41598-024-68659-2)
Supplement: Supplementary file 1 — Supplementary Figure 1. [file 41598_2024_68659_MOESM1_ESM.pdf]

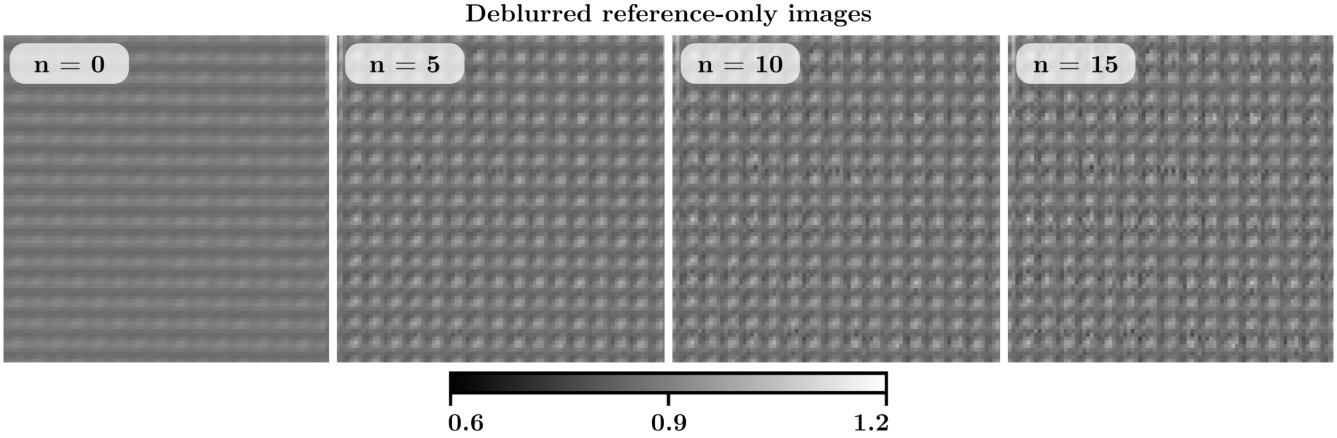

Supplementary Figure 1. Magnified region of the reference-only image from the SPring-8 data set deblurred using the position-dependent deblurring operator with a different number of iterations n. The optimal number of iterations was chosen to be $n = 10$ as this deblurred the image without overamplifying the image noise.
